# NLRP3 Inflammasome: The Stormy Link Between Obesity and COVID-19

**DOI:** 10.3389/fimmu.2020.570251

**Published:** 2020-10-30

**Authors:** Alberto López-Reyes, Carlos Martinez-Armenta, Rocio Espinosa-Velázquez, Paola Vázquez-Cárdenas, Marlid Cruz-Ramos, Berenice Palacios-Gonzalez, Luis Enrique Gomez-Quiroz, Gabriela Angélica Martínez-Nava

**Affiliations:** ^1^ Laboratorio de Gerociencias, Instituto Nacional de Rehabilitación Luis Guillermo Ibarra Ibarra, Secretaría de Salud, Ciudad de México, México; ^2^ Facultad de Ciencias de la Salud, Universidad Anáhuac, Ciudad de México, México; ^3^ Postgrado en Biología Experimental, Dirección de Ciencias Biológicas y de la Salud (DCBS), Universidad Autónoma Metropolitana Iztapalapa, Ciudad de México, México; ^4^ Centro de Innovación Médica Aplicada, Hospital General Dr. Manuel Gea González, Ciudad de México, México; ^5^ Cátedras de Consejo Nacional de Ciencia y Tecnología (CONACYT), Instituto Nacional de Cancerología, Ciudad de México, México; ^6^ Unidad de Vinculación Científica de la Facultad de Medicina, Universidad Nacional Autónoma de México (UNAM), Instituto Nacional de Medicina Genómica, Ciudad de México, México; ^7^ Laboratorio de Fisiología Celular, Departamento de Ciencias de la Salud, Universidad Autónoma Metropolitana Iztapalapa, Ciudad de México, México; ^8^ Laboratorio de Líquido Sinovial, Instituto Nacional de Rehabilitación Luis Guillermo Ibarra Ibarra, Ciudad de México, México

**Keywords:** severe acute respiratory syndrome coronavirus 2, coronavirus disease 2019, pyroptosis, obesity, inflammasome

## Abstract

Several countries around the world have faced an important obesity challenge for the past four decades as the result of an obesogenic environment. This disease has a multifactorial origin and it is associated with multiple comorbidities including type 2 diabetes, hypertension, osteoarthritis, metabolic syndrome, cancer, and dyslipidemia. With regard to dyslipidemia, hypertriglyceridemia is a well-known activator of the NLRP3 inflammasome, triggering adipokines and cytokines secretion which in addition induce a systemic inflammatory state that provides an adequate scenario for infections, particularly those mediated by viruses such as HIV, H1N1 influenza, and SARS-CoV-2. The SARS-CoV-2 infection causes the coronavirus disease 2019 (COVID-19) and it is responsible for the pandemic that we are currently living. COVID-19 causes an aggressive immune response known as cytokine release syndrome or cytokine storm that causes multiorgan failure and in most cases leads to death. In the present work, we aimed to review the molecular mechanisms by which obesity-associated systemic inflammation could cause a more severe clinical presentation of COVID-19. The SARS-CoV-2 infection could potentiate or accelerate the pre-existing systemic inflammatory state of individuals with obesity, *via* the NLRP3 inflammasome activation and the release of pro-inflammatory cytokines from cells trough Gasdermin-pores commonly found in cell death by pyroptosis.

## Introduction

Obesity has reached epidemic proportions globally; thus, the World Health Organization (WHO) identifies it as a serious public health problem, particularly in west countries (1). The global increase of obesity in the last 50 years has doubled, and it has been estimated that a third of the world population is obese or overweight (2). The WHO defines obesity as a complex entity in which there is an excessive accumulation of fat that affects practically all body functions and compromises the individual’s health (1). Furthermore, obesity is considered the fifth risk factor for mortality, as it is the main risk factor of diabetes, cardiovascular disease, hypertension, dyslipidemia, musculoskeletal disorders such as osteoarthritis, and other diseases (3–5). It is well known that obesity leads to a low-grade chronic inflammation promoted by the release of adipokines and cytokines (6). This dysfunctional state contributes to a systemic lipotoxicity affecting liver, muscle and pancreas, activating NOD-, LRR-, and the pyrin domain-containing protein 3 (NLRP3) inflammasome (7–10). Beyond cellular damage, organ dysfunction and metabolic compromise, the low-grade chronic inflammation could condition to viral diseases, such as those instigated by HIV (11, 12), H1N1 influenza virus (13–15), and SARS-CoV-2 (16).

The severe acute respiratory syndrome coronavirus 2 (SARS-CoV-2) causes the coronavirus disease (COVID-19), which is responsible for more than 758,942 deaths worldwide (17). The WHO has listed COVID-19 as a global health crisis (18). Similar to obesity, this virus induces a systemic inflammation; however, the SARS-CoV-2 produces an uncontrolled increase of cytokine secretion causing multiple organ failure, followed by death (19–21). It should be noted that during the cytokine storm caused by SARS-CoV-2, the inflammasome could be involved in the maintenance of inflammation, as it happens in obesity (22–24). Given that obesity might be associated with the development of aggressive clinical symptoms in COVID-19, we aimed to suggest the possible role of NLRP3 inflammasome as a link between obesity and the increased risk for a severe COVID-19 outcome.

## Inflammasome and Pyroptosis

The NLRP3 inflammasome is a multiprotein complex present in macrophages, dendritic cells and other non-immune cells. The activation of NLRP3 as the pivotal component of the innate immune system, plays a critical role in the host defense against bacteria, fungi and viruses among others; however, the NLRP3 is also associated with metabolic and inflammatory conditions such as gout, diabetes mellitus, insulin resistance, and obesity (25–28).

**Figure 1:**
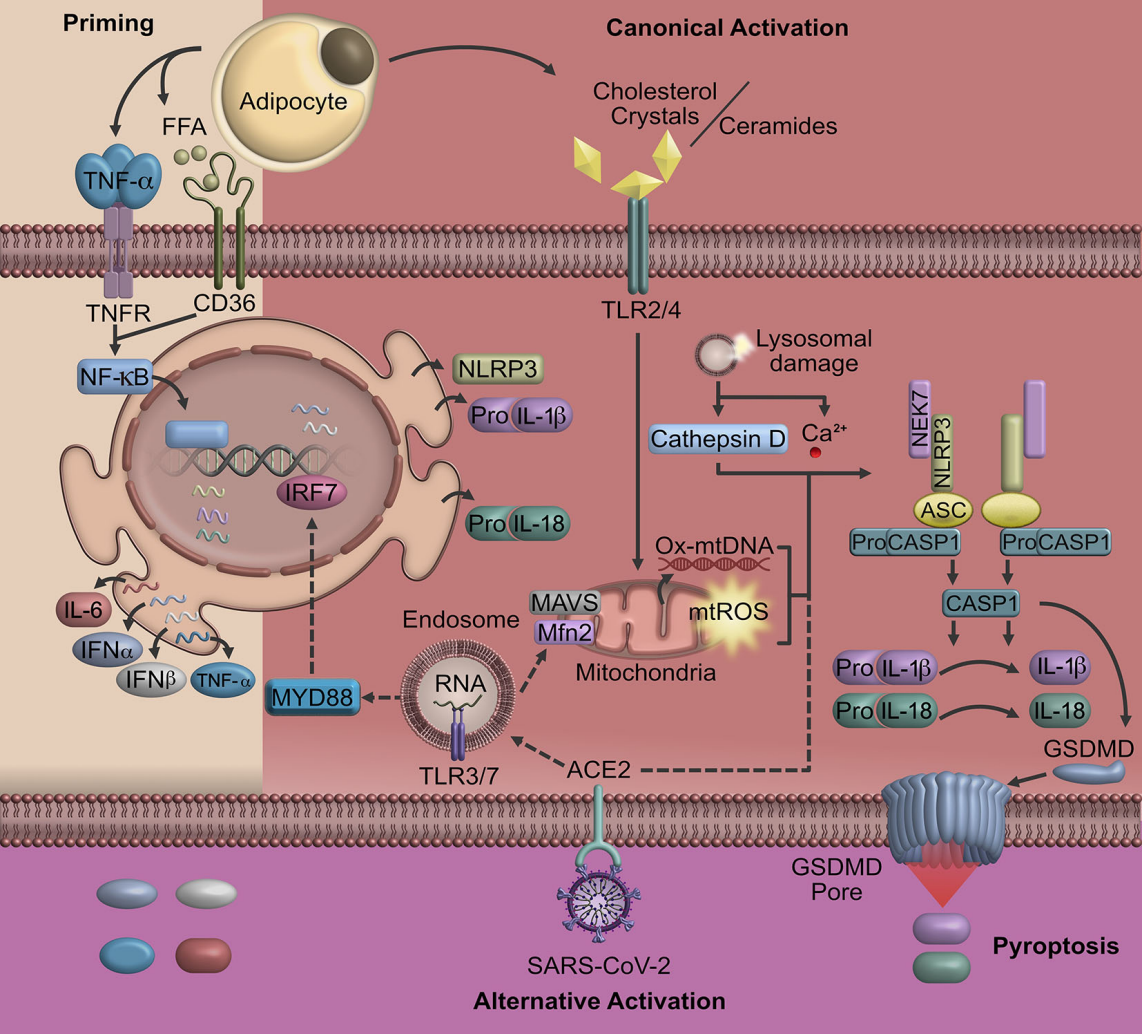
Pyroptosis triggered in obesity and COVID-19. A possible signaling that triggers the activation of NLRP3 and consequently cell pyroptosis in COVID-19 may be linked to obesity. In individuals with obesity, pyroptosis is characterized by the activation of NOD-like receptors that induce the formation of cell membrane pores mediated by Gasdermin D and the release of inflammatory factors. The SARS-CoV-2 uses ACE2, a receptor highly express in AT, to entry human host cells promoting the expression of pro-inflammatory cytokines and oligomerization of NLRP3. Upon the NLRP3 inflammasome activation, Gasdermin-pores and cell membrane swelling promote cell pyroptosis, particularly in macrophages and lymphocytes. Cell signaling represented by straight lines show the canonical activation of NLRP3 in obesity. Dotted line represents the possible contribution of SARS-CoV-2 in NLRP3 activation. ACE2, angiotensin-converting enzyme 2; AT, adipose tissue; Mfn2, mitofusin-2; MAVS, mitochondrial antiviral signaling protein; GSDMD, Gasdermin D; IRF, interferon-regulatory factor; ROS, reactive oxygen species; TLR, toll-like receptor; SARS-CoV-2, severe acute respiratory syndrome coronavirus 2; TRIF, protein-inducing interferon-β.

The inflammasome is coordinated by the NLRP3 sensor [Nucleotide-Binding Oligomerization Domain (NOD), Leucin-rich repeat (LRR), Pyrin domain (PYD), adaptor protein ASC, as well as the effector protein caspase 1] (29, 30). In most cases, the activation of NLRP3 is regulated by pathogen-associated molecular patterns (PAMPs) and damage-associated molecular patterns (DAMPs) that are recognized by the Toll-like receptors (29, 31). The canonical activation of NLRP3 requires two independent signals; an initial priming signal and a second one to be fully activated (**Figure 1**).

The priming signal begins when PAMPs and other inflammatory mediators such as interleukin 1-beta (IL-1β) and tumor necrosis factor-alpha (TNF-α) bind to their respective receptors (Pattern Recognition Receptors, IL-1βR, TNF-αR). These receptors induce the activation of nuclear factor kappa-light-chain-enhancer of activated B cells (NF-κB), which promotes the transcription of NF-κB-dependent genes such as NLRP3, pro-IL-1β, and pro-interleukin-18 (IL-18) (31–33). NF-κB also modulates the gene expression of interleukin-6 (IL-6), interleukin-8 (IL-8), interleukin-12 (IL-12), TNF-α, and interferon-gamma inducible protein 10 (IP-10) (31, 34). These cytokines play a critical role in acute inflammation, as well as in promoting the synthesis of acute phase proteins that modify permeability and endothelial functions leading to the recruitment of other immune system cells. In particular, TNF-α and IL-6 regulate transcription and transduction of IL-1β (34).

The second activation signal of NLRP3 could be initiated by several extracellular stimulus including those mediated by crystals of cholesterol, uric acid, asbestos and β amyloid, which induce the release of ROS (reactive oxygen species), as well as lysosomal enzyme cathepsin B and Ca^2+^ caused by a destabilization and rupture of lysosome or endosome (35–37). The increase of cathepsin B and Ca^2+^ as well as aberrant ionic flux may trigger mitochondrial damage. Mitochondrial dysfunction occurs *via* an increase of mitochondrial ROS, oxidized mitochondrial DNA, cardiolipins and proteins that respond to viruses such as mitofusin-1 and -2 or mitochondrial antiviral signaling protein, promoting the oligomerization of NLRP3 (25, 38). This assembly allows caspase-1 to start the cleavage of pro-IL-1β and pro-IL-18 (**Figure 1**).

The final step of the NLRP3 inflammasome activation is the cleavage of Gasdermin D (GSDMD) by caspase-1, resulting in the release of GSDMD N-terminal fragments that are essential for pore formation on cell membranes (39). These pores lead to the release of cytosolic content causing unrestrained dissemination of inflammatory mediators including IL-1β, IL-18, that induce cell death by pyroptosis contributing to host immune defense (40, 41).

## Obesity, Inflammasome, and Pyroptosis

As a functional organ, adipose tissue (AT) is the main endocrine and immunological tissue implicated in the pathophysiology of obesity and metabolic dysfunction (42, 43). AT has a complex and heterogeneous composition that includes endothelium, extracellular proteins matrix, stem cells, fibroblasts and immune cells, and adipocytes (43–46). There are three types of AT. White AT is the organ responsible for storing energy mainly in the form of triglycerides for energy demand periods. In contrast, brown AT is composed of rich mitochondria and multivacuolar smaller adipocytes that are positive for the expression of uncoupling protein-1, which is responsible for thermogenesis and generation of heat rather than ATP from the oxidation of fatty acids (47). Finally, the most recently AT identified, beige adipocyte tissue, resembles BAT morphology and function, it can differentiate from precursors found in WAT in response to stimuli as cold exposure (48–50).

In obesity, white AT promotes cellular, molecular and biochemical alterations that cause local and systemic changes. Locally, adipocyte hyperplasia and hypertrophy modify the AT structure; at a systemic level, these alterations promote inflammation, insulin resistance, nonalcoholic fatty liver diseases, and dyslipidemias (42, 45, 51, 52).

The impaired adipocytes function caused by hyperplasia and hypertrophy induce an exacerbated lipolysis releasing fatty acids (such as palmitic and lauric acids), and triggers the formation of ceramides and cholesterol crystals that activate tissue-resident macrophages through the TLR4 signaling (53–55). This macrophage stimulation itself triggers the production of ROS, calcium accumulation, as well as the release of IL-6, TNF-α, and monocyte chemoattractant protein-1 (MCP-1) (56–58). The chemokine MCP-1 leads to the recruitment of monocytes, while the interferon gamma (IFN-*γ*), secreted by T cells in AT stimulates a polarization process in macrophages, from the anti-inflammatory state (M2) to the pro-inflammatory (M1) phenotype thereby perpetuating a low-grade systemic inflammation (56, 57, 59, 60).

The inflammatory activity of M1 macrophages is traditionally mediated by the activation of TNF-αR, IL-1βR, and CD36, which is a priming signaling that activates NF-κB promoting the transcription of NLRP3, pro-IL-1β, pro-IL-18, and other inflammatory cytokines (61). However, for the NLRP3 inflammasome assembly, a second hit is required; this second hit is induced by the binding of ceramides, fatty acids, oxidized low-density lipoproteins and cholesterol crystals to TLR 2/4 (37, 58, 62–64). Finally, the sustained activation of NLRP3 will induce the assembly of GSDMD pores into the macrophage cell membrane (40, 65). This process constitutes the pyroptotic cell death mechanism, disrupting the osmotic potential and pouring pro-inflammatory molecules to the system (65) (**Figure 1**).

## COVID-19, Inflammasome, and Pyroptosis

The causal agent of COVID-19 is known as SARS-CoV-2. The viral infection is mediated by the attachment between a spike glycoprotein and the angiotensin-converting enzyme 2 (ACE2) in human host cells (66). The host target receptor mediates a virus-cell membrane fusion and a viral entry that could cause virus-linked pyroptosis (67, 68), leading to SARS-CoV-2-induced lymphopenia (22, 69).

The innate immune system cells detect the viral RNA by Pattern Recognition Receptors like TLR 3/7 in the endosome; then, cascades of signaling pathways are triggered by TRIF and MyD88 leading to the activation of transcription factors including NF-κB and interferon-regulatory factor 3/7 (IRF) (70). Not only TLR signaling can induce an excessive inflammatory response to SARS-CoV-2, the inflammasome activation stimulated by viral internalization can also induce it; presumably, this occurs through spike proteins binding to CD147 (71). The massive release of TNF-α, IFN-*γ*, IL-1β, IL-8, MCP-1, and IP-10 seen in acute phase of COVID-19 patients (22) may probably be linked to pyroptosis, especially in lymphocytes through the NLRP3 inflammasome activation.

The pyroptosis-mediated cell death has been described previously in another coronavirus infection (72, 73). Recent evidence suggests that Severe Acute Respiratory Syndrome-related Coronavirus (SARS-CoV) induces NLRP3-dependent pyroptosis in macrophages, which is triggered by the essential ion channel activity of viroporin 3a (72) as well as by a direct interaction of ORF8b with the LRR domain of NLRP3 (73). Moreover, it has been demonstrated that ORF3a and E protein can stimulate NF-κB signaling, resulting in the transcription of NLRP3, chemokines, and pro-inflammatory cytokine, including IL-1β, IL-18, and IL-8 (74–76). Additionally, ORF3a might also mediate NLRP3 inflammasome activation through the ubiquitination of ASC promoting maturation and secretion of IL-1β (75). In contrast, E protein induces the assembly of NLRP3 inflammasome *via* the formation of pores in Endoplasmic Reticulum-Golgi intermediate compartment membranes that triggers a massive calcium ion transportation to the cytosol (77–79). Finally, Chang et al. highlight the biological role of SARS-CoV unique domain (SUD) as a direct inductor of NLRP3 inflammasome activation in alveolar epithelial cells, as well as its activity modulating pulmonary inflammation mediated by CXCL10 *in vitro* and *in vivo* through NLRP3 inflammasome pathway (80). These molecular mechanisms have been linked to the induction of cytokine storm and cell death in SARS-CoV (72, 73, 81, 82).

In the particular case of SARS-CoV-2 strains, recent evidence suggests similar signaling pathways with SARS-CoV in modulating the inflammation by activating NLRP3. A novel study has revealed homology functional domains of ORF3a when compared with those reported in SARS-CoV strains, suggesting some hypothetical pathways of ORF3a linked to the NF-κB activation and NLRP3 inflammasome assembly (83). Considering SARS-CoV-2 has high nucleotide sequence homology to SARS-CoV and 94.7% amino acid identity of E protein (84, 85), it could be inferred that pyroptosis might play a central role in the pathogenesis of COVID-19. Individuals infected with SARS-CoV-2 often show high concentration of pro-inflammatory cytokines (22, 86), which is a downstream indicator of inflammatory programmed cell death (**Figure 1**) (65).

Different reports have shown that COVID-19 is characterized by a dysfunctional immune response, which exacerbates the disease progression as result of a persistent inflammation associated with high peripheral levels of IL-1β, IL-6, TNF-α, MCP-1, and IP10 (22, 87–89). This aggravated inflammatory response triggers a cytokine storm, contributing to the pathological inflammation and multi-organ injury seen in severely ill COVID-19 patients (23, 90–92). With regard to the exacerbated inflammation probably caused by aberrant activation of NLRP3 inflammasome in COVID-19, potential targets are being explored including host signaling proteins and effector molecules that lead cytokine storm (24, 93–95).

To date, some different drugs such as Acalabrutinib have shown their beneficial effects in COVID-19. This therapeutic strategy inhibits the Bruton tyrosine kinase (BTK) enzyme, which is a direct regulator in NLRP3 inflammasome activation (96, 97). The use of the drug in severe COVID-19 patients showed a decrease in serum inflammatory biomarkers (24). Furthermore, Colchicine (93–95) has been successfully tested as inhibitor of NLRP3 inflammasome, improving survival outcomes in COVID-19 patients since it suppresses caspase-1 activation and subsequent IL-1β and IL-18 processing (94, 98). In this sense, Hydroxychloroquine, another NLRP3 inhibitor, has shown a role affecting the NLRP3 inflammasome activation and assembly (99–101). In addition, different clinical trials registered to evaluate the efficacy of pharmacological inhibitors of the NLRP3 inflammasome in treating COVID-19; include Colchicine (NCT04326790, NCT04322565, NCT04328480, NCT04322682), Hydroxychloroquine in combination with Azithromycin (NCT04339816, NCT04336332), Melatonin (NCT04409522), and Tranilast (ChiCTR2000030002); versus standard care.

In other hand, recent studies have evaluated the use of Anakinra (102, 103) as a therapeutic strategy focused in signaling inhibition of IL-1β to treat COVID-19 related cytokine storm. This drug is a human IL-1β receptor antagonist that inhibits inflammation response. Moreover, there are different clinical trials registered to study the efficacy and safety of Canakinumab (anti-IL-1β monoclonal antibody) in COVID-19-induce pneumonia (NCT04362813, NCT04348448) and COVID-19 cardiac injury (NCT04365153). In addition, Tocilizumab (anti-IL-6 treatment) showed clinical improvement in COVID-19 patients (104–106). There are few drugs with mechanisms of action targeting NLRP3 such as Necrosulfonamide (107) and Disulfiram (108), by inhibiting the N-terminal GSDMD pores. The NLRP3 inhibition may represent an optimal strategy to mitigate the impact of comorbidities associated with COVID-19 such as diabetes mellitus (109, 110), hypertension (111), and obesity (112, 113).

## The Clinical Impact of Obesity in COVID-19

Different studies have reported fatal COVID-19 outcomes in individuals with at least one chronic disease such as hypertension, diabetes, cardiovascular disease, and obesity (87, 114–116). In individuals infected with SARS-CoV-2, overweight and obesity could be conditioning the critical outcome of COVID-19. The high number of young individuals with COVID-19 that have been hospitalized might be explained by the high obesity incidence found among them (117–119). Furthermore, data suggest that overweight and obesity determined by BMI are associated with the presence of severe pneumonia or increased incidence of ICU admission of individuals with COVID-19 in the USA (117, 120, 121), China (122, 123), Mexico (114, 124), and France (125).

A possible explanation is that tissue expression of ACE2 may play a key role in the progression of COVID-19 patients with obesity, since obese individuals have increased AT mass that leads to an elevated number of ACE2-expressing cells and therefore an increased risk of SARS-CoV-2 infection (126, 127). Other obesity-implicated conditions have been associated with a severe course of COVID-19 such as respiratory symptoms, impaired metabolic health, cardiac stress, dysfunctional host defense against viral infection, and multi-organ damage (120, 125).

**Figure 2:**
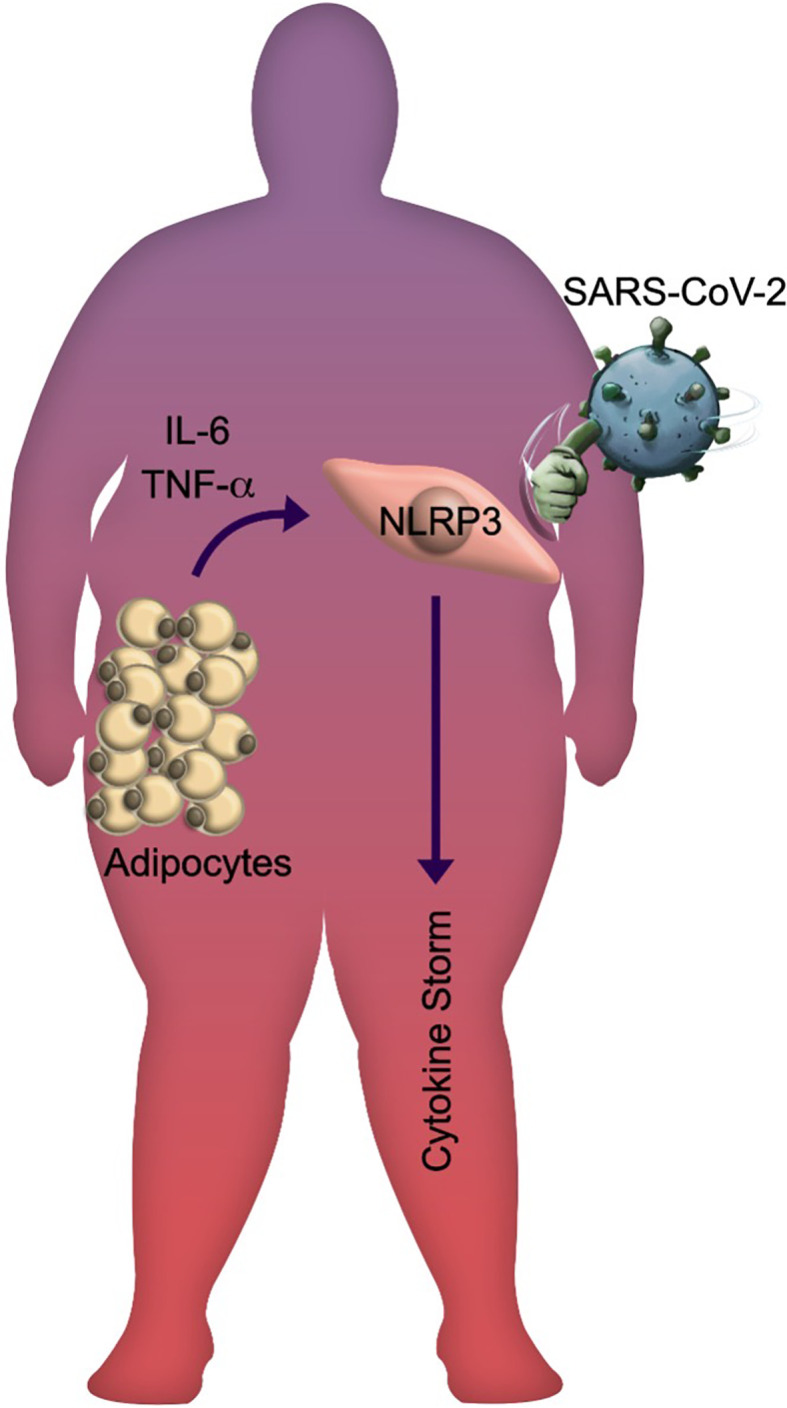
The SARS-CoV-2 knockouts obese individuals. Schematic representation of the lethal impact generated by SARS-CoV-2 infection in obese individuals with metabolic and inflammatory stress. The released adipokines by hypertrophic and hyperplasic adipose tissue promote the NLRP3 inflammasome activation, leading to systemic inflammation that is amplified during viral infection.

Interestingly, obesity has been associated with a decrease in mortality in patients with acute respiratory distress syndrome (ARDS), and this is referred to as the “obesity paradox”. However, the high mortality among patients with obesity who manifest SARS-CoV-2 infection has prompted the notion that SARS-CoV-2 has disproved the “obesity paradox” in ARDS (128, 129). The paradox fades if body composition (i.e., fat mass, lean mass, and skeletal muscle mass), body fat distribution (abdominal obesity carries a higher risk of developing metabolic disorders than peripheral or gluteofemoral obesity), AT functionality, and the differences between subcutaneous and visceral AT are considered (130–135). Moreover, the metabolically unhealthy obese phenotype seems to be associated with increased activation of the NLPR3 in macrophages infiltrating visceral AT and a less favorable inflammatory profile than the metabolically healthy phenotype (136). In obese individuals, the innate immune system might be already in a “primed state” due to chronic low-grade inflammation and this could promote an hyperinflammatory response (137, 138); under this scenario, we wonder if trained immunity mediated by NLRP3 in obese conditions to severe outcomes in COVID-19 patients, or normal weight patients infected by SARS-CoV-2 are developing trained immunity that accelerates and trigger short-term development of degenerative chronic comorbidities such as atherosclerosis, diabetes, osteoarthritis, gout, autoimmune diseases, and even obesity itself. This sustained activation state could induce poor clinical outcomes of COVID-19, amplifying the pro-inflammatory response to SARS-CoV-2 infection (**Figure 2**).

## Discussion

Recent evidence suggests that 1) AT hypertrophy and hyperplasia promote the synthesis of triglycerides, oxidized phospholipids, IL-1β, TNF-α, and adipokines triggering the systemic inflammatory state regularly observed in individuals with obesity (53–55); 2) these molecules are responsible for increasing the vulnerability to infections in individuals with obesity because the priming phase of the inflammasome is already active (61); 3) the recognition of SARS-CoV-2 by endosomal TLRs 3/7 will trigger multiple signaling and cellular pathways that will allow inflammasome assembly and consequent maturation of cytosolic pro-cytokines such as IL-1β and IL-18 as well as the activation of GSDMD (70, 83); 4) the Gasdermin-pore formation will start cell death by pyroptosis *via* the release of pro-inflammatory mediators in COVID-19 patients (22, 83, 86). To summarize the recognition of the molecular pathways involved in the inflammasome might explain the vulnerability of obese patients to develop severe cases of COVID-19 (**Figure 2**).

## Author Contributions

AL-R developed the concept for the review. MC-R, CM-A, and RE-V wrote the manuscript. PV-C, LG-Q, BP-G, and GN contributed to the editing and contextual design. All authors contributed to the article and approved the submitted version.

## Funding

Financial support: CONACYT 312513 SARS-COV 2.

## Conflict of Interest

The authors declare that the research was conducted in the absence of any commercial or financial relationships that could be construed as a potential conflict of interest.
